# Administration and burden of subcutaneous immunotherapy for allergic rhinitis in clinical practice in Canada

**DOI:** 10.1186/1710-1492-10-S1-A10

**Published:** 2014-03-03

**Authors:** Steven W Blume, Karen Yeomans, Harold Kim, Sunning Tao, Stephanie M Hubbard, Felicia Allen-Ramey

**Affiliations:** 1 Evidera, Bethesda, MD, 20814, USA; 2 United BioSource Corporation, Montreal, QC, H9S 5J9, Canada; 3 United BioSource Corporation, Lexington, MA, 02420, USA; 4 Merck & Co., Inc., West Point, PA, 19486, USA

## Background

Allergic rhinitis (AR) has been estimated to affect approximately 20–25% of Canadians. [1] Management of AR encompasses allergen avoidance, use of symptomatic medications, and allergen immunotherapy for patients unresponsive to other pharmacotherapy. [2,3] This study was conducted to characterize patients receiving subcutaneous immunotherapy (SCIT) and the SCIT administration process in Canada and the United States; Canadian results are presented.

## Methods

A multi-center, prospective, observational study was conducted at 5 allergy clinics in Quebec and Ontario and 1 primary care clinic in Quebec from March-September 2012. Patients ≥6 years who were scheduled for SCIT on study days were invited to participate in the study. Patients enrolled in a clinical trial, receiving sublingual immunotherapy or allergic only to insect venom, latex, food, or drugs were excluded. Site and patient-specific information were captured via direct observation, questionnaires, and medical chart review. Costs were estimated from time and supply observation and query.

## Results

A total of 294 patients were enrolled with a mean age of 44 years (4% <18 years and 9% ≥65 years). Of these, 59% were female, 81% Caucasian, 57% employed full-time and 30% reported household income ≥$100,000. Concomitant allergy medications were reported by 66% of patients; 25% used asthma medications. Two-thirds of patients reported initiating SCIT because they desired a cure “once and for all” for their allergies. Primary symptoms at initiation of SCIT were nasal congestion (62%), rhinorrhea (59%), sneezing (35%) and itchy eyes (32%). Chart data indicated that patients received treatment for several different antigens (mean: 4; SD: 3); those most commonly noted were ragweed (82%), house dust mites (55%), grass (48%) and tree (48%). Sites reported a SCIT build-up phase requiring one injection/week over 12-52 weeks. The SCIT maintenance phase was reported as one injection/month over 4-5 years. Site-specific means (SDs) for total patient time in the clinic for SCIT ranged from 32 (11) to 49 (10) minutes, including a 30-minute required post-injection observation at all but one site. Average patient travel time to the office for SCIT was 20 (SD: 14) minutes. Mean time missed from work in the previous week was 0.7 hours. The direct costs of an injection ranged from $14 to $41 by site, with extract preparation or acquisition and administrative tasks the largest components.

## Conclusions

Patients initiated SCIT to permanently resolve allergy symptoms. SCIT requires a long-term commitment, resulting in considerable direct and indirect costs.
